# METTL13 facilitates cell growth and metastasis in gastric cancer via an eEF1A/HN1L positive feedback circuit

**DOI:** 10.1007/s12079-022-00687-x

**Published:** 2022-08-04

**Authors:** Qiong Wu, Qingqing Hu, Yanan Hai, Yandong Li, Yong Gao

**Affiliations:** 1 Department of Oncology, Shanghai East Hospital, School of Medicine, Tongji University, 150 Ji-Mo Rd., Shanghai, 200120 China; 2 Research Center for Translational Medicine, Shanghai East Hospital, School of Medicine, Tongji University, Shanghai, 200120 China

**Keywords:** METTL13, Gastric cancer, Metastasis, HN1L, eEF1A

## Abstract

Although improved treatment could inhibit progression of gastric cancer (GC), the recurrence and metastasis remain challenging issues. Methyltransferase like 13 (METTL13) has been implicated in most human cancers, but its function and mechanism in GC remain elusive. In the present study, we evaluated its expression in GC samples and found it was aberrantly overexpressed in cancer tissues than that in normal stomach tissues. High expression of METTL13 was closely associated with age, tumor size and T classification. Biological experiments showed that silencing METTL13 suppressed gastric cancer cell proliferation and metastasis in vivo and vitro, whereas opposite effects were observed upon METTL13 overexpression. Further mechanistic explorations revealed that METTL13 regulated the expression of HN1L (Hematological and neurological expressed 1-like), which is reported to be an oncogene in various cancers. Knockdown of HN1L dampened gastric cancer cell growth induced by METTL13. Eukaryotic translation elongation factor-1A (eEF1A), the present sole methylation substrate of METTL13, was involved in the regulation of HN1L by METTL13 in a K55 methylation independent manner. In addition, we also found HN1L could facilitate METTL13 expression in GC cells consistent with a previous report in hepatocellular carcinoma. Thus, these findings demonstrate a METTL13/eEF1A/HN1L positive feedback circuit promoting gastric cancer development and metastasis. It will help develop promising diagnostic and therapeutic targets for this disease.

**Supplementary Information:**

The online version contains supplementary material available at 10.1007/s12079-022-00687-x.

## Introduction

Gastric cancer (GC), originates from gastric mucosa, is one of major malignancies with high incidence, mortality and lacks of effective treatments worldwide (Chen et al. 2016; Liu et al. 2019a; Siegel et al. 2019). Despite advanced surgical techniques, chemotherapy, molecular-targeted therapy and oncology immunotherapy that could inhibit development and metastasis of GC, the overall 5-year survival rate is still low (Siegel et al. 2017; Torre et al. 2016). The main character of GC is relatively rapid progression and late clinical presentation, accounting for the increased mortality and morbidity of patients (Han et al. 2019). It would be prevented largely if the disease is identified at the early stages. Therefore, it’s urgent to identify available robust diagnostic biomarker and comprehend the mechanism governing gastric cancer.

METTL13 (methyltransferase like 13, also named FEAT), located at 1q24.3, was initially identified from rat brain as a suppressor of nuclear apoptosis (Nagase et al. 1998). Several studies have revealed that METTL13 influences occurrence of multiple diseases including cancer. METTL13 was found to be upregulated in liver, breast, lung and pancreatic cancer, which was significantly associated with patient poor survival. A METTL13 transgenic mouse model confirmed its oncogenic role in vivo. Recently, Liu et al. demonstrated that METTL13-mediated eEF1A (Eukaryotic translation elongation factor-1A) K55 methylation facilitated tumorigenicity via regulating protein synthesis. Another research in lung and pancreatic cancer patient-derived xenograft models further indicated that METTL13 depletion markedly inhibited KRAS-driven pancreatic tumorigenesis (Liu et al. 2019b). More recently, METTL13 has been shown as a crucial mediator of HN1L (hematological and neurological expressed 1-like) in modulating hepatocellular carcinoma (HCC) cell growth and metastasis via co-activating TCF3 (transcription factor 3) and ZEB1 (zinc finger E-box binding homeobox 1) expression (Li et al. 2019). Although METTL13 has been implicated to be an important oncogene in various cancers, some studies also demonstrated its tumor suppressive function in bladder cancer and clear cell renal cell carcinoma (Liu et al. 2021; Zhang et al. 2016). These evidences suggest that METTL13 may play divergent roles in different tissue types. As a potential target for cancer therapy, it is necessary to fully explore the role and underlying mechanism of METTL13 in tumorigenesis. However to date, the functional significance and expression pattern of METTL13 in GC has not yet been elucidated.

In the current study, we aimed to detect the expression and functional roles of METTL13 in GC tissues and cell lines. METTL13 was upregulated in most gastric cancer tissues compared to the corresponding adjacent normal stomach tissues by qRT-PCR and immunohistochemistry analysis. Functional experiments showed that METTL13 played oncogenic roles in cell proliferation and migration of GC cells. Moreover, we found eEF1A, the methylation substrate of METTL13, mediated the regulation of METTL13 on HN1L, an oncogene encoding a 190-aa protein, which in turn activated METTL13 expression, forming a METTL13/eEF1A/HN1L regulatory loop to promote GC development and progression.

## Materials and methods

### Clinical samples

To examine METTL13 mRNA expression in GC, 48 pairs of gastric cancer samples were collected from diagnosed GC patients during surgical resection in Shanghai East Hospital. All the specimens were frozen in liquid nitrogen immediately after resection and then stored at − 80 °C fridge for subsequent experiments. In order to detect METTL13 expression at protein level in GC, a tissue microarray construction (TMA, Cat# HStmA180su11, containing 90 tumor tissues and 90 corresponding normal tissues) was purchased from Shanghai Outdo Biotech Co.,Itd and analyzed by two pathologists who are unknown about clinical information of patients after immunohistochemical staining. The staining results were classified as previously described (Wu et al. 2018). In brief, 0–100% was scored by positive cell percentage and 0–3 was scored on the basis of staining intensity. According to the product of percentage of positive cells and staining intensity: negative (0–0.5); slight positive (0.5–1.5); moderate positive (1.5–2.5); strong positive (≥ 2.5). Anti-METTL13 antibody (#ab186002, Abcam, Cambridge, MA, USA) was used at a dilution of 1:4000 for immunohistochemistry. The use of clinical samples and this study were approved by the Ethics Committee of Shanghai East Hospital, Tongji University School of Medicine. Written informed consent was obtained from each patient involved in this study.

### Cell lines and cell culture

All the gastric cell lines (BGC823, AGS, MGC803, SGC7901, MKN28) were obtained from Shanghai Cell Bank of the Chinese Academy of Sciences (Shanghai, China), which were maintained in DMEM (Dulbecco’s modified Eagle’s, Corning, Inc, Corning, NY, USA) medium supplemented with 10% fetal bovine serum (FBS) and 1% penicillin/streptomycin (M&C Gene Technology Ltd, Beijing, China). The cells were cultured in a humidified incubator with 5% CO_2_ at 37 °C.

### RNA extraction and qRT-PCR assay

We used TRI Reagent to separate total RNAs from GC cells or human tissues (Sigma-Al-drich, MO, USA) referred to manufacturer’s instructions. First-strand cDNA were synthesized with PrimeScript RT reagent Kit, Takara, Kyoto, Japan. Analysis of qRT-PCR was performed on an ABI QuantStudio 6 Flex Real- time PCR system (Applied Biosystems, CA, USA) with TB Green Premix Ex Taq II (Takara, Kyoto, Japan). All primers used in the experiments are summarized in the Supplementary Table 1. 2^−ΔΔCt^ method was utilized to assess relative fold change compared to the control samples. β-actin worked as the endogenous reference.

### Transfection and lentivirus transduction

SiRNAs targeting METTL13, HN1L, eEF1A1 or eEF1A2 and control siRNA were synthesized by Gene Pharma (Shanghai, China). The target sequences are listed in the Supplementary Table 1. A full-length METTL13 or HN1L cDNA was cloned into pcDNA 3.1 mammalian expression vector respectively. EEF1A expression plasmids, pEnter-eEF1A1 and pEnter-eEF1A2 were purchased from Vigene Biosciences, Shandong, China. Lipofectamine 3000 (Invitrogen, Thermo Fisher Scientific, US) was applied to transfect siRNAs or plasmids according to the manufacturer’s instructions. To establish the cells stably overexpressing METTL13, SGC7901 cells were transfected pcDNA3.1-METTL13 plasmid and pcDNA3.1 vector, respectively. Then cells were selected by G418 (900 μg/ml). Single colony was isolated and amplified. Western blot analysis was used to screen METTL13 overexpressed colonies. Lentiviral particles for silencing METTL13 (Lv-shMETTL13 and Lv-shNC based on siMETTL13-1 and si-NC sequence) were packaged by Genepharma (Shanghai, China). Stably infected cells were selected by puromycin (2 μg/ml) after transducing cells with lentiviral particles.

### Western blot assay

Total proteins were extracted from cultured GC cells with RIPA buffer, and the supernatants were diluted in 5×SDS loading buffer after calculating protein concentration with a Pierce BCA protein assay kit (Takara, Japan). Western blot assay was carried out in accordance with the previous report (Yu et al. 2019). The signal bands were detected by Odyssey Infrared Imaging System (Li-COR Biosciences) based on manufacturer's instructions. The primary antibodies used in this study are as follows: METTL13 (#ab186002, Abcam), HN1L (#ab200571, Abcam), eEF1A (#2551, Cell Signaling Technology), β-actin (#81178, Santa Cruz Biotechnology).

### Cellular proliferation assay

To evaluate GC cell viability, cell counting kit-8 assay was performed (CCK-8 reagent, Dojindo, Japan). In brief, 3000–5000 cells/well were seeded into 96-well plates in triplicates. 10 μl of CCK-8 solution was added into wells at scheduled time intervals (0 h, 24 h, 48 h, 72 h and 96 h). Optical density value at 450 nm was measured on an automated microplate reader (SpectraMax M5, Molecular Devices, USA) following 37 °C incubation for 1 h. For colony formation assay, 1500 GC cells were inoculated in 6-well plates per well. Two weeks later, colonies were fixed with 4% paraformaldehyde for 10 min. Then rinsed and stained with 0.5% crystal violet for 10 min at least. The number of colonies were calculated using Image J software (v1.52, National Institutes of Health Freeware, USA). The experiments were performed in triplicate and repeated at least three times independently.

### Transwell migration assay

Modified 8 μm 24 well Boyden chambers (pore size; Costar; Corning, Inc.) were applied to evaluate cell migratory ability. In brief, 5 × 10^4^ cells were seeded into upper chambers supplemented 400 μl of DMEM without FBS in triplicates. The bottom chambers were filled 800 μl DMEM containing 10% FBS. After cultured 24 h, cells on surface of bottom were stained with crystal violet and calculated under microscope. The experiments were repeated as least three times independently.

### Wound healing assay

Cells stably overexpressing or knocking down METTL13 and corresponding control cells were collected and then planted into a 6-well plate in triplicates. A yellow plastic tip was used to scratch the cell monolayer (90% confluence). Then rinsed cells twice with 1× PBS and cultivated in serum-free DMEM medium for up to 48 h culture. The wound gap was photographed at 0 h, 24 h and 48 h under the light microscope (Nikon, Japan) with 200× magnification.

### Animal experiments

About 4–5 weeks old male BALB/c nude mice were provided by Sippr-BK laboratory animal corporation (Shanghai, China). To establish subcutaneous xenograft model, all mice were divided into two groups (n = 5 or n = 6 per group). 2 × 10^6^ stably infected cells (BGC823-LV-shNC, BGC823-LV-shMETTL13) or stable cells lines (SGC7901-Vec, SGC7901-METTL13-15#) were collected and implanted into nude mice by subcutaneous injection at the site of the dorsal flank. Tumors were weighed and photographed 4 weeks later. For tumor metastasis assay in vivo, 1.5 × 10^6^ cells were injected into each mouse by tail vein, respectively (n = 5 per group). After five weeks, mice were sacrificed and tumor nodules on lung surface were calculated. In addition, lung tissues were fixed with 4% paraformaldehyde and hematoxylin and eosin (H&E) staining was performed. All animal handling and experimental procedures used in this study were approved by the Ethics Committee of Shanghai East Hospital.

### Datasets

The public data in this study were obtained from Co-expedia database (http://www.coexpedia.org/) (Yang et al. 2017) and Gene Expression Profiling Interactive Analysis (GEPIA, http://gepia.cancer-pku.cn/) based on The Cancer Genome Atlas (TCGA) database (Tang et al. 2017). Genes co-expressed with METTL13 in gastric cancer were examined using Co-expedia database. Through GEPIA online tools (http://gepia.cancer-pku.cn/), we analyzed the correlation between HN1L and METTL13 expression in gastric cancer.

### Statistical analysis

Statistical analysis for three independent experiments was analyzed by GraphPad Prism 8. The quantitative data was represented as mean ± SD. Student *t* test and one-way ANOVA were performed to calculate the difference comparison. The χ^2^ test was performed to analyze the relationships between METTL13 expression and a series of clinicopathological characteristics. *P* < 0.05 was considered to be significant.

## Results

### METTL13 was frequently increased in gastric cancer

**Fig. 1:**
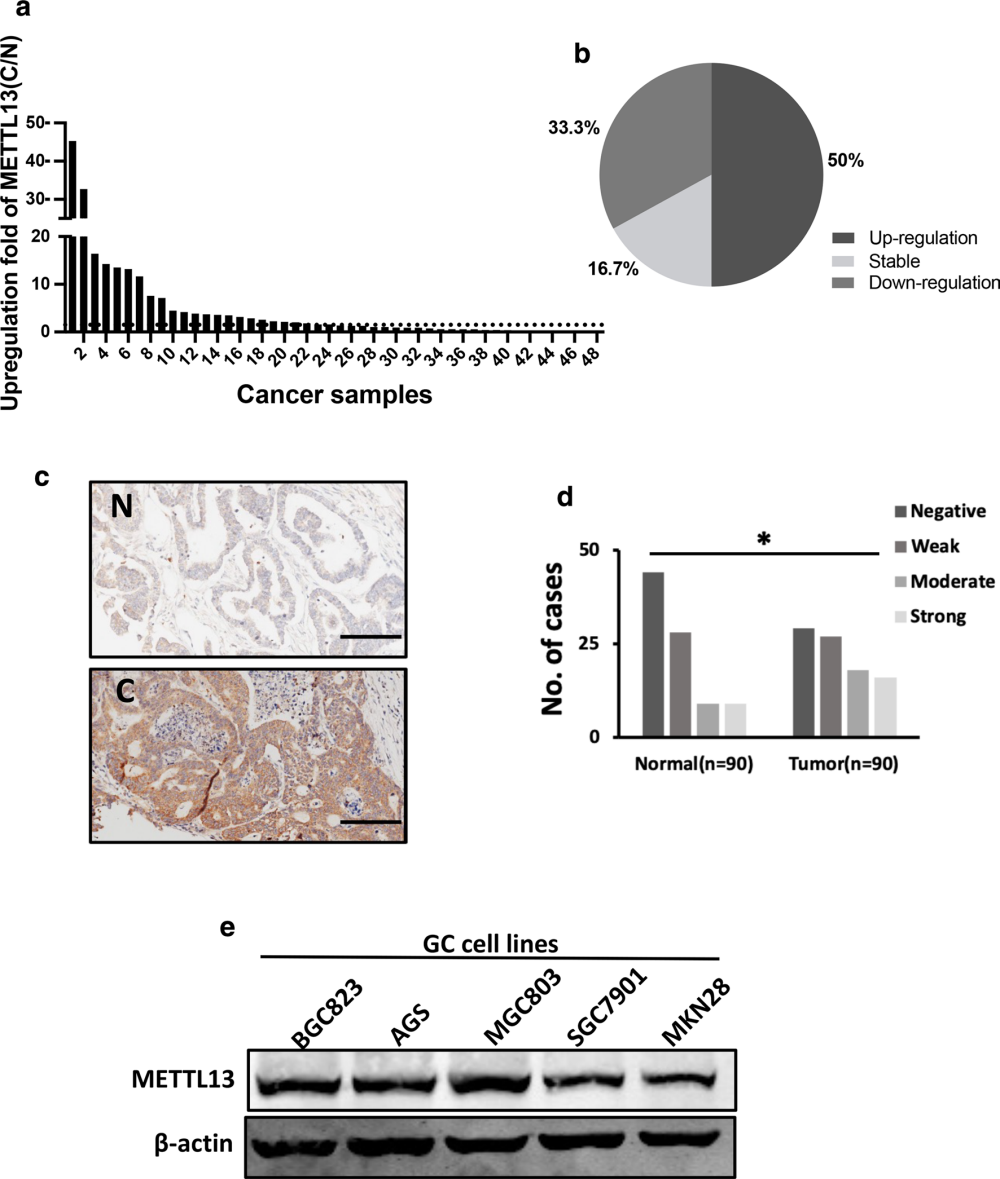
METTL13 was aberrantly elevated in gastric cancer. **a** METTL13 expression levels examined by qRT-PCR in 48 pairs of GC tissues and adjacent non-cancer tissues. Data are presented as relative > 1.5 folds change (C/N: C, cancer tissues; N, non-cancer tissues). **b** Percentage of METTL13 expression alteration in 48 paired of GC samples. Upregulation: C/N > 1.5; Downregulation: C/N < 1.5. **c** A pair of representative GC sample with IHC staining was shown. Scale bar:100 μm. **d** Statistical analysis of METTL13 staining strength in a tissue microarray containing 90 pairs of GC samples. *Chi*-square test was used. *P < 0.05. **e** Western blot analysis was performed to detect protein expression of METTL13 in five kinds of GC cell lines

To investigate METTL13 expression in GC, we collected 48 pairs of human gastric cancer samples and paired normal adjacent gastric tissues from diagnosed patients and examined the expression of METTL13 via qRT-PCR. The results showed that METTL13 mRNA level was significantly elevated in the GC tissues compared to corresponding normal tissues, and 24/48 (50%) exhibited more than 1.5-fold increase (Fig. 1a, b). Here, we are of the opinion that more than 1.5-fold increase is remarkable and classified into up-regulated group, less than 0.667-fold change is downregulated and (0.667–1.5)-fold change is not obvious or no change, which is classified as stable group. Next, we utilized IHC (immunohistochemical staining) to detect METTL13 protein expression in a tissue microarray consisting of 90 tumor samples and 90 non-tumor tissues. Consistent with the qRT-PCR result, increased METTL13 protein expression was also observed in GC tissues relative to the matched non-tumor tissues (Fig. 1c, d). Meanwhile, the correlation between METTL13 expression and clinicopathological characteristics of patients was analyzed and summarized in Table 1. High METTL13 expression was significantly correlated with age (*P* = 0.005), tumor size (*P* < 0.001) and T classification (*P* = 0.027), but not correlated with other clinicopathological features, such as gender, N status and M status. These collective findings indicated that METTL13 plays potential important role in gastric cancer development.

**Table 1:** The correlation of METTL13 expression with clinicopathological features of gastric cancer patients

	Number of cases (n = 90)	Negative, weak (n = 56)	Moderate, strong (n = 34)	*P* value
Age (years)				
> 50	32	26	6	0.005*
≤ 50	58	30	28	
Gender				
Male	62	37	25	0.458
Female	28	19	9	
Tumor size				
≤ 5 cm	9	3	6	< 0.001*
> 5 cm	81	53	28	
T classification				
T1, T2	9	4	5	0.027*
T3, T4	81	52	29	
N status				
N0	26	15	11	0.572
N1–3	64	41	23	
Metastasis				
M0	88	56	32	0.136
M1	2	0	2	
Stage				
I, II	33	20	13	0.809
III, IV	57	36	21	

*P < 0.05 was considered as a significant association among the variables

### METTL13 knockdown suppressed GC cell growth in vitro and in vivo

**Fig. 2:**
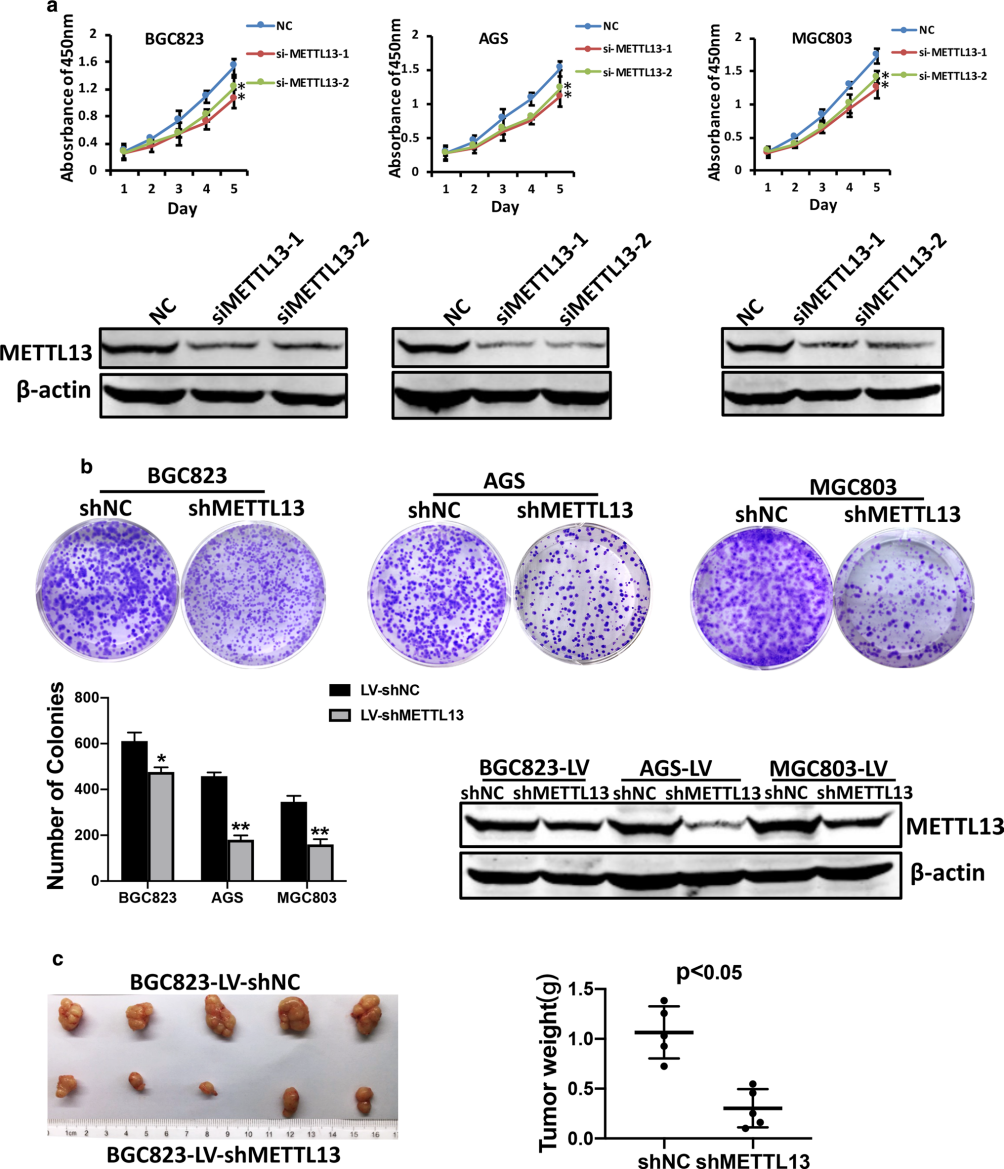
Downregulation of METTL13 inhibited GC cell proliferation in vivo and in vitro. **a** CCK-8 assay was performed to evaluate cell viability in BGC823, AGS and MGC803 cells transfected with siMETTL13-1, siMETTL13-2 and control respectively. Knockdown efficacy of METTL13 was confirmed by western blot analysis. **b** Colony formation abilities were carried out in BGC823, AGS and MGC803 cells with stable knockdown of METTL13. Western blot results were shown to indicate the knockdown efficacy in these cells as indicated. **c** After 4 weeks subcutaneous injection of BGC823-LV-shNC and BGC823-LV-shMETTL13 cells in animal model, mice were sacrificed and then tumors were weighed. All data are given as mean ± SD. *P < 0.05; **P < 0.01

To evaluate the role of METTL13 in gastric cancer, we detected the protein expression of METTL13 in five kinds of GC cell lines by western blotting (Fig. 1e). Three kinds of cell lines (BGC823, AGS and MGC803) that had relatively high METTL13 expression were selected for knockdown experiments whereas two cell lines (BGC823 and SGC7901) that had lower amount of METTL13 were for overexpression experiments. We firstly introduced siRNAs targeting METTL13 into BGC823, AGS and MGC803 cells by transient transfection. Knockdown efficacy was confirmed by western blot analysis (Fig. 2a). In CCK-8 cell viability assay, METTL13 knockdown caused an obvious inhibition on cell growth (Fig. 2a). Next, we generated METTL13-silenced stable cell lines by infection with lentivirus particles in the three GC cell lines. Colony formation assay demonstrated that fewer and smaller colonies were observed in LV-shMETTL13 cells compared to the control cells (Fig. 2b). Moreover, METTL13 knockdown markedly inhibited tumorigenicity of BGC823 cells and decreased tumor weight in nude mice (Fig. 2c). These results above suggested that silencing METTL13 strongly suppresses gastric cancer cell proliferation in vitro and in vivo.

### Downregulation of METTL13 attenuated GC cell motility

**Fig. 3:**
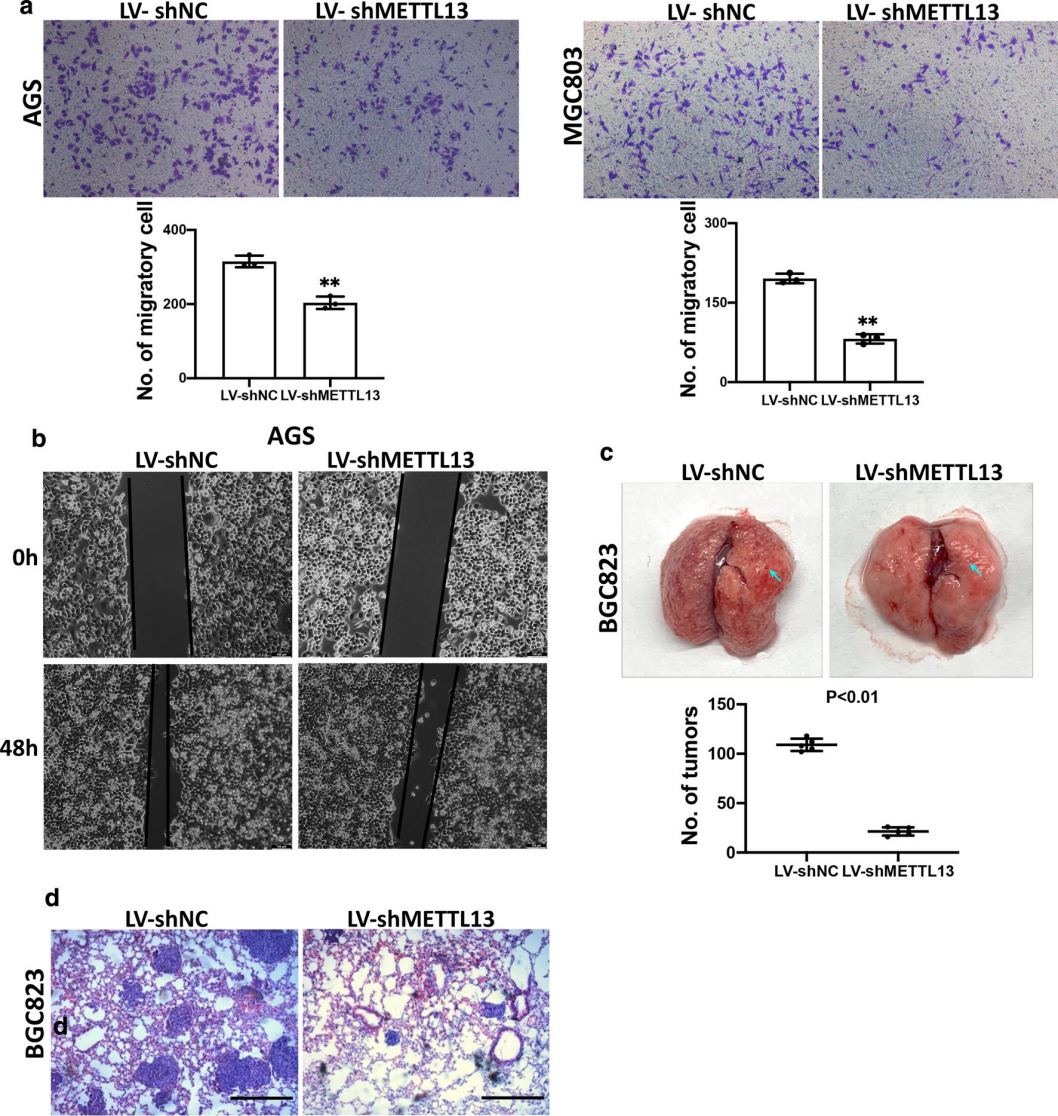
METTL13 knockdown attenuated cell migration and pulmonary metastasis. **a** Transwell migration assay was performed in METTL13 silenced GC cells and control cells indicated. **b** Wound healing assay was employed to investigate motility ability of GC cells with METTL13 knockdown. **c** Pulmonary metastatic foci were observed on lung surface of mice (n = 5 per group), representative lung pictures were shown. The number of metastatic loci in lung surface was counted. **d** Metastatic lung tissues were stained with H&E staining (hematoxylin and eosin). Scale bar:100 μm. Data are given as mean ± SD. *P < 0.05; **P < 0.01

To determine the potential effect of METTL13 on GC cell migration, transwell assay was carried out. The results indicated that migratory cells were evidently decreased in METTL13-silenced AGS and MGC803 cells compared to control cells (Fig. 3a). Concordantly, METTL13 knockdown significantly diminished the motility distance of AGS cell with scratched wound healing assay (Fig. 3b). In addition, pulmonary metastasis assays by tail vein injection were performed to examine whether METTH13 affects GC cell metastasis in vivo. As expected, decreased metastatic foci number on lung surfaces was observed in METTL13 knockdown group than those in control group (Fig. 3c). Histological analysis further confirmed the metastatic foci on the lung surface of mice (Fig. 3d). These data demonstrated that METTL13 knockdown suppressed the migration and motility of GC cells.

### Overexpression of METTL13 promoted GC cell growth and migration

**Fig. 4:**
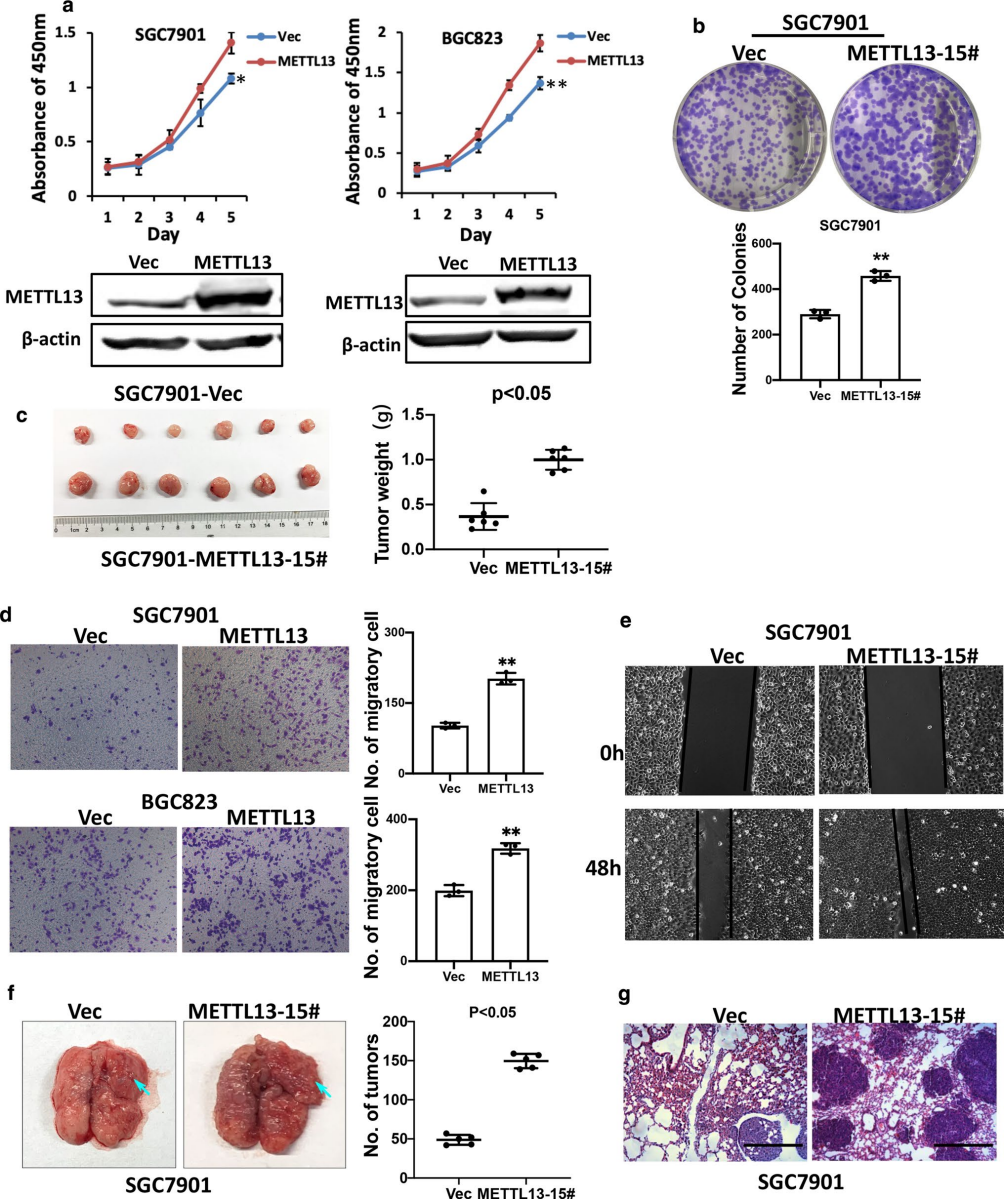
Upregulation of METTL13 positively regulated cell proliferation and metastasis. **a** The influence of METTL13 overexpression on cell viability in BGC823 and SGC7901 cells via CCK-8 assays. Western blot was used to examine the expression of METTL13 in GC cells indicated. **b** Colony formation experiments were conducted in SGC7901 cells with stable METTL13 overexpression. **c** Overexpression of METTL13 promoted xenograft tumor formation in nude mice model. **d** METTL13 overexpression in SGC7901 cells enhanced cell migration in transwell assay. **e** Wound healing assay revealed METTL13 enhanced cell motility of SGC7901 cells. **f** Increased number and size of metastatic nodules on the lung surface were observed in the METTL13 overexpressed group than the control group after 55 weeks by tail veins injection (n = 5 per group). **g** Representative images of HE staining (hematoxylin and eosin) were shown. Data are given as mean ± SD (*P < 0.05; **P < 0.01)

To confirm the effects induced by METTL13 knockdown, we performed overexpression experiments to elucidate the function of METTL13 in GC cells. Enhanced expression of METTL13 by transient transfection with pcDNA3.1-METTL13 plasmid promoted GC cell viability in CCK-8 assay (Fig. 4a). To gain a stable overexpression of METTL13 in SGC7901 cells, we used a traditional limiting dilution method to screen the colonies with METTL13 overexpression and finally SGC7901-METTL13-15# was successfully isolated. Colony formation assays showed this overexpression cell line had strong capacity to promote cell growth (Fig. 4b). In a mouse xenograft model, METTL13 overexpression also resulted in larger tumor size and tumor weight (Fig. 4c). On the other hand, transwell migration and wound healing assay results demonstrated that enhanced METTL13 expression in SGC7901 cells accelerated cell migratory compared to those control cells (Fig. 4d, e). To further verify the effect of METTL13 overexpression in metastasis, we employed pulmonary metastasis mice model by tail vein injection of SGC7901-Vec and SGC7901-METTL13-15# cells. An obvious increase in the number and size of metastatic nodules on the lung surface was observed in the METTL13-overexpressed group after 5 weeks injection (Fig. 4f). The histological assessment on the lung metastatic nodules was also performed (Fig. 4g). These findings suggested that METTL13 overexpression in GC cells plays oncogenic roles.

### METTL13 co-expressed with HN1L and modulated HN1L expression in GC

**Fig. 5:**
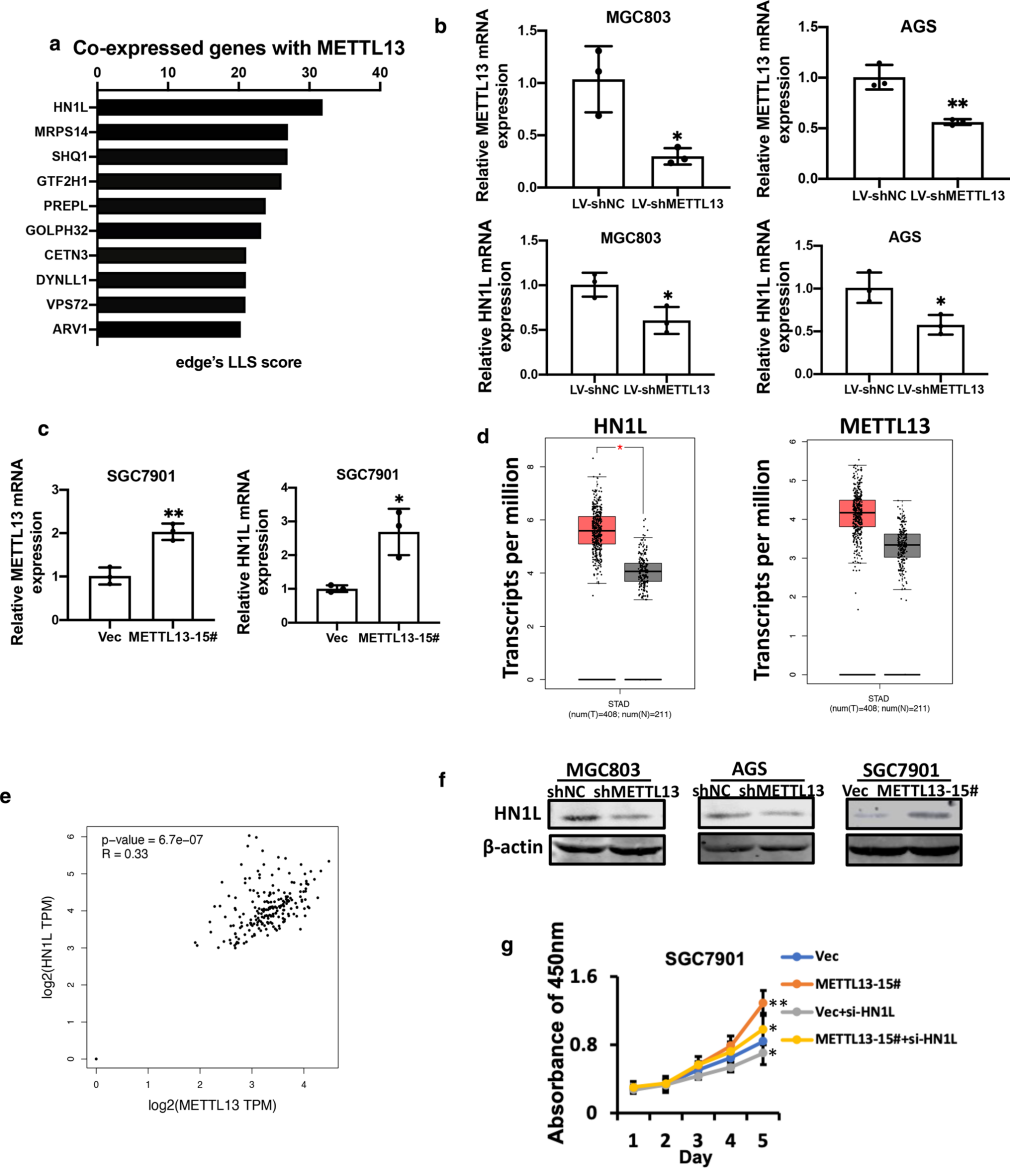
METTL13 modulated HN1L expression in GC. **a** Genes that showed positively association with METTL13 were surveyed by COEXPEDIA database (http://www.coexpedia.org/). qRT-PCR was performed to test the expression of METTL13 and HN1L in METTL13 knockdown cells (**b**) and METTL13 overexpression cells (**c**). **d** HN1L or METTL13 mRNA expression was upregulated in GC tissues than that in the normal tissues based on GEPIA online analysis for TCGA database. **e** Positive correlation between METTL13 and HN1L expression at mRNA levels were analyzed using GEPIA online tools. **f** Western blotting was applied to test HN1L protein level upon METTL13 knockdown or overexpression in GC cells. **g** CCK-8 assays showed that cell viability caused by overexpression of METTL13 was partially attenuated by introducing siRNA targeting HN1L into SCG7901 cells. Data are given as mean ± SD. *P < 0.05; **P < 0.01

To understand the molecular mechanisms by which METTL13 promotes GC cell growth and metastasis, we surveyed genes positively co-expression that showed functional association with METTL13 in human by Co-expedia database. Co-expressed genes with METTL13 were shown in Table 2. The top ten genes differentially co-expressed with METTL13 were listed based on analysis results (Fig. 5a). Then, we tested the top three gene expression at mRNA level upon upregulating or downregulating METTL13 in GC cell lines. Interestingly, HN1L expression obviously decreased in METTL13 knockdown cells (BGC823 and AGS), while increased in METTL13 overexpression cells (SGC7901) (Fig. 5b, c), indicating that HN1L could be a target gene of METTL13 in GC. However, MRPS14 or SHQ1 expression was not consistent after silencing METTL13 with shRNA in BGC823 and AGS cells (Supplementary Fig. 1a). HN1L (Hematological and neurological expressed 1-like), also known as L11, is a homologous gene of HN1 encoding a 190-aa protein (Ko et al. 2000; Varisli et al. 2012). Previous reports indicated that HN1L was overexpressed in various cancer types and involved in tumor proliferation and metastasis (Li et al. 2017; Liu et al. 2018; Petroziello et al. 2004; Zhou et al. 2004). CCK-8 assay was performed and results showed that silencing HN1L reduced GC cell viability, whereas overexpression of HN1L enhanced the effect (Supplementary Fig. 1b).

**Table 2:** The co-expression partners of METTL13

Gene	Co-expression partners
*METTL13*	HN1L,MRPS14,SHQ1,GTF2H1,PREPL,GOLPH3L,CETN3,DYNLL1,VPS72,ARV1,TRIM27,DNAJA3,SRSF1,GART,AHSA1,C14orf119,ACTL6A,FASTKD1,COA3,DCAF7,SF3B3,SEC23IP,GIN1,KRCC1,TXNDC9,TRMT1L,GIMAP4,VPS35,NDUFAF1,CRNKL1,COPB2,ATG4C,R,F20,GEMIN5,RAB10,NOC3L,ZC3H13,ELAVL1,GIMAP6,IDH1,ELP3,TIMMDC1,CCT5,CSE1L,DCP1B,WDR3,UTP14C,NUDCD1,FARSA,MRS2,TMEM251,CHCHD4,UMPS,PAICS,PAQR8,COQ9,EXOSC3,FBXO22,C1GALT1C1,RRP15,PEX11B,FAM114A2,WDR12,GEMIN6,ARFIP1,MAT2B,MCCC2,EIF2S1,LCLAT1,AARS,G3BP1,DENND2D,C4orf27,LARS2,MRPS31,ISG20L2,VAMP4,GIMAP1,NBR1,PHF14,PARP1,PCMT1,C17orf80,NSL1,LRIF1,LDAH,ERI2,RNASEL,HIBCH,DYNC1I2,TTI1,TMCO1,LARP4,COX15,TBCK,DDX23,MED11,CAD,RUVBL1,MKKS,MRPS28,SLC35A5,DPY30,VKORC1L1,GIMAP8,SPOP,BRI3BP,POGK,RPE,LYRM7,SLC35B3,SUCLA2,HEATR1,KEAP1,IRF2,PROSC,INTS7,PIGC,THNSL1,PUS7,SUV420H1,HSD17B4,OMA1,NDUFA8,ICAM2,ACTR3,SNX1,PMS1,EHBP1,ARMT1,THYN1,ZKSCAN1,PDCD11,ASH2L,COQ5,RMI1,BET1,NUP37,ATP6V1D,FANCF,COG2,PPIL1,STX7,GGPS1,NCOR1,MITD1,OGFOD1,MAT2A,PNPT1,PIGF,RPA2,C1orf43,PRRC2C,COG6,GPALPP1,IFNAR1,ZNF260,GLT8D1,MTO1,ST8SIA4,NEK7,HLTF,CHTOP,MINPP1,CPOX,SLC33A1,FPGT,NUP43,FLAD1,CMTR2,NHP2L1,NDUFS2,FAHD1,TNFAIP8L2,ZNF566,BBS10,PCNA,KATNBL1,GLS,TRIM5,ORMDL2,GOLGA7,TIMM21,USO1,CX3CR1,FAM118B,MRPL18,PHF10,TAF5,POT1,IARS2,PDHX,MOSPD1,TBCE,VTA1,RPA1,MRPL9,BRD8,ALDH18A1,AHCYL1,CSRP2BP,TP53,KIAA0907,MRAS,IPO8,NAT10,GTF2E1,CBR4,GINS1,HNRNPF,AIMP2,COA4,MBTPS2,SLC35A1,UBE2Q1,INTS5,TMCO6,HMGCL,MEN1,ZADH2,ZC3H4,MRPL35,CCR2,DLAT,LMBRD2,OSGEPL1,ST6GAL1,MAPK1IP1L,AP5M1,FBXO9,CBX1,PUM2,TIA1,FPR3,SERINC3,EMG1,NLRC4,SNRNP35,XPO5,CLCN3,YTHDF2,GEMIN2,PPP1R21,ZNF189,CLPX,SON,TRMT10C,KIF2A,MYCBP,NR1H3,PIGU,DDX1,KCTD21,BLZF1,MINA,ZSCAN26,SELPLG,MTERF3,DIEXF,KLHL12,FAM98B,TMEM168,SOS1,NOL10,TBL2,C11orf68,DCAF12,PI4KB,GMPS,MCM3,CLK2,VPS36,BFAR,OAS2,DCAF10,HARS,DLGAP5,M6PR,ECT2,ISOC2,DTL,PAFAH1B1,EIF4EBP1,RBM8A,MORC2,ZNF146,MARS,UBTD2,IPO13,ZBED8,KLHDC10,RRP1B,INSRR,WIPF1,IPO11,SEC23B,C2orf42,E2F6,FBXO5,RPP40,ECI2,PSMD14,ICMT,CCT7,NCBP1,TAF2,ZMYM6,MRPL51,CSTF3,COG5,MPHOSPH8,NMNAT1,PRUNE,SLC26A2,ALS2,HPS5,WDR77,ENSA,TXLNG,ANAPC10,CFAP97,POLR3KORC2,TRMT61B,NUDC,CCT3,PSMB4,PSMD3,POLR1C,UTP6,SF3B4,MRPS18B,POLD3,SNAPIN,NEK2,TIPRL,SRP68,WDR81,ABHD6,MON2,IPO9,USP21,TMEM69,CARD8,GTF3A,RFT1,RDX,SYNRG,DSCC1,MRPL27,FAM111A,FBXO28,RAB3GAP2,C9orf69,HERC4,FAM98A,RBM4B,C19orf52,SMC1A,TJP2,STIP1,SLC30A6,CENPF,PRDX3,PANK1,KIF4A,CPM,PA2G4,RSBN1L,RAD51C,LYAR,HSBP1,GMPR2,CENPL,CHORDC1,PNO1,ATP6V1B2,ZNF691,DHX33,ATP6V1C1,SNAPC5,C11orf54,TMEM203,FAM220A,CRLS1,CDK1,PDE12,GRWD1,YY1AP1,SCYL3,FTSJ2,CEP162,UBAP2L,C5orf51,MFAP1,TRMT6,PDHB,EIF2B2,RFC4,MGAT2,SNUPN,GNPAT,NOLC1,CACYBP,F11R,SRPK1,HMMR,ZNF140,CCDC85B,CRKL,LACTB2,BANF1,CCDC51,NUP133,TOR1A,UFC1,CCNB2,ADRM1,ELP4,MRPL46,PAK2,HAX1,CREBL2,TRAPPC6B,PRCC,EXOSC5,C1orf27,SH3BP4,SDHC,EMC2,SGK223,LMAN2L,IMP3,SNAPC3,METTL5,RPP25L,LAMTOR2,TMEM126B,PFKM,HSP9AB1,TMEM184C,TMEM218,PRKACB,GOSR2,TRNT1,MAD2L1,PSMA4,NME7,IVNS1ABP,MRPL19,UGDH,POLR1B,FAM192A,MRPS12,TRMT10A,CUEDC2,RRP7A,ACOT13,METTL18,PRIMPOL,B4GALT3,DESI2,ING4,TFCP2,PANK2,CNOT1,PRMT5,GMNN,SRSF8,UTP14A,POLR3B,CDC20,CKS1B,PRRC1,SLC25A19,KIF20A,WDR4,RBM12,BCCIP,PRKAR1A,WDR92,CDKN3,ABCF2,NIP7,NDC80,PYCR2,ANAPC7,RNF5,DNTTIP2,ELOVL6,PTPRK,PPP2CB

We hypothesized that METTL13 could contribute to GC cell proliferation and metastasis by regulating HN1L expression. To test this hypothesis, we analyzed METTL13 or HN1L mRNA expression using transcripts per million (TPM) data based on TCGA database which consists of 408 gastric tumor and 211 normal gastric tissue by GEPIA online tool. As is shown in Fig. 5d, HN1L and METTL13 mRNA expression were upregulated in GC tissues than that in normal tissues separately. Simultaneously, a positive correlation between METTL13 and HN1L expression was also obtained from GEPIA online tool based on TCGA database (Fig. 5e). We further explored HN1L expression by qRT-PCR in the previous mentioned 48 pairs of clinical GC samples. The results strongly proved that HN1L expression was remarkably elevated in GC tissues (Supplementary Fig. 1c) and positively correlated with METTL13 level (Supplementary Fig. 1d). Consistent with the qRT-PCR results, HN1L protein expression was changed with METTL13 increase or decrease (Fig. 5f). To verify the functional link between HN1L and METTL13, we transiently silenced HN1L expression in METT13 overexpression cells and control cells. The results of CCK-8 assays revealed that HN1L downregulation attenuated METTL13-induced cell viability (Fig. 5g), suggesting HN1L may function as downstream effector of METTL13 in GC.

### eEF1A mediated the expression regulation of HN1L by METTL13 in a K55 methylation independent manner

**Fig. 6:**
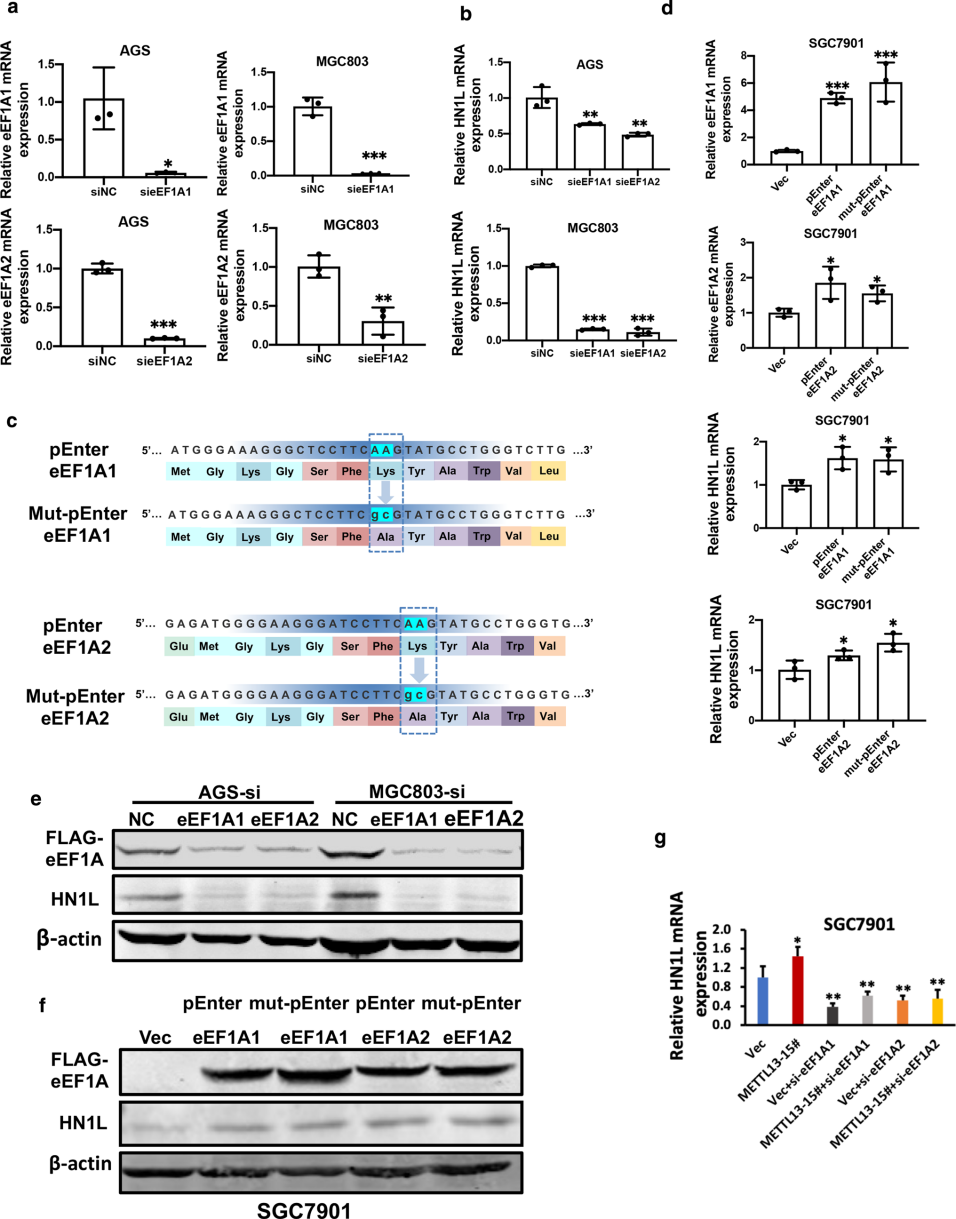
eEF1A was involved in the regulation of HN1L by METTL13. **a** Knockdown efficacy for eEF1A1 or eEF1A2 was verified by qRT-PCR in AGS and MGC803 cells. **b** HN1L expression was analyzed by qRT-PCR in eEF1A1 or eEF1A2 knockdown cells. **c** Mutation site of eEF1A1/eEF1A2. **d** mRNA level of eEF1A1 or eEF1A2 was tested by qRT-PCR in SGC7901 cells transfected with pENTER-eEF1A1 or pENTER-eEF1A2 plasmid. And overexpression of eEF1A1 or eEF1A2 led to expression upregulation of HN1L by qRT-PCR analysis. No obvious change of HN1L expression was observed between wild type eEF1A and eEF1A K55A mutant cells. **e** Western blotting indicated that HN1L was decreased when eEF1A1 or eEF1A2 was silenced. **f** Western blot results were shown, indicating both wild-type and K55A mutant eEF1A upregulated HN1L expression. **g** Knockdown of eEF1A could partially offset the upregulation of HN1L in METTL13-overexpressed cells. Data are given as mean ± SD. *P < 0.05; **P < 0.01

Eukaryotic translation elongation factor-1A (eEF1A) belongs to GTP-binding protein which has two closely related homologues, eEF1A1 and eEF1A2. They have 90% sequence identity (Abbas et al. 2015). Recently, eEF1A was identified as a substrate of METTL13, which methylates eEF1A at lysine 55. METTL13 promotes protein synthesis in tumorigenicity via eEF1A methylation (Liu et al. 2019b). To examine whether METTL13 regulates HN1L to influence GC progression via eEF1A, two separated siRNAs against eEF1A1 and eEF1A2 were transfected into AGS and MGC803 cells, respectively. qRT-PCR results indicated that HN1L expression was significantly downregulated with eEF1A knockdown (Fig. 6a, b). Conversely, exogenous expression eEF1A1 and eEF1A2 could upregulate HN1L expression (Fig. 6d). Furthermore, we mutated the K55 to A55 of eEF1A (Fig. 6c). Western blot results confirmed the regulation of HN1L by eEF1A (Fig. 6e, f). Importantly, the K55 mutation to A55 of eEF1A had no obvious effect on HN1L expression (Fig. 6c–f), implying that the methylation modification of eEF1A by METTL13 was not indispensable for activating HN1L expression. Statistical analysis for Fig. 6f was shown in the Supplementary Fig. 2a. In addition, eEF1A1 or eEF1A2 knockdown abolished the upregulation of HN1L caused by METTL13 overexpression (Fig. 6g). These results implicated that METTL13 promotes HN1L expression in an eEF1A/K55 methylation-independent manner.

Considering that little is known about the roles of eEF1A1 and eEF1A2 in GC, we elucidated their roles on cell growth via CCK-8 assay. The results showed that downregulated eEF1A1 or eEF1A2 suppressed GC cell viability, whereas upregulated eEF1A1 or eEF1A2 promoted opposite effects (Supplementary Fig. 2b, c), hinting that eEF1A plays oncogenic roles in GC, in line with the roles of METTL13 and HN1L. Based on a previous report in live cancer indicating that HN1L could positively regulate METTL13 expression, we tested whether this regulation relationship exists in GC. The final qRT-PCR results proved that HN1L promoted METTL13 expression as well in GC (Supplementary Fig. 2c). Collectively, our data notably advocated that METTL13 could regulate HN1L expression through eEF1A in an eEF1A/K55 methylation independent manner, while HN1L in turn, activates METTL13 expression.

## Discussion

To our best knowledge, gastric cancer cells are characterized by uncontrolled proliferation and metastasis (Zhang et al. 2020). On account of occult and atypical symptoms in the early stage, the vast majority of GC patients are diagnosed at an advanced or late stage. Despite of advanced therapeutic treatments, survival rate of GC patients was still poor. Therefore, identifying genes as biomarkers and exploring molecular mechanism underlying GC progression and metastasis remain as major challenges (An et al. 2018; Mayer et al. 2014; Zhou et al. 2019). In this context, our study indicated the expression of function of a tumor-promoting gene, METTL13, in GC for the first time.

METTL13, a member of N-terminal protein methyltransferase family, has been implicated to regulate lysine 55 methylation of eEF1A protein. The two proteins are known to contribute to tumorigenesis in several cancer types. In our GC samples, we showed that both mRNA and protein expression of METTL13 were elevated compared to the corresponding non-tumor tissues. Statistical analysis revealed an obvious relationship between METTL13 and clinicopathological characteristics, such as age, tumor size and T classification, suggesting that METTL13 could be a potential biomarker for GC. Given that the increased expression of METTL13 in cancer tissues, the role of METTL13 in gastric cancer were subsequently investigated. The results of in vitro experiments showed that gastric cancer cells proliferation and migration ability were promoted following METTL13 overexpression. Furthermore, we established mice models for the functional investigations of METTL13 in vivo and demonstrated that METTL13 facilitated GC proliferation and metastasis. These findings are in agreement with the above analyzed clinical data and other cancer reports that METTL13 promotes tumorigenesis.

By Co-expedia database, we found METTL13 and HN1L have a close expression correlation in clinical samples. HN1L belongs to hematological and neurological expressed 1 family. Studies have showed that HN1L is upregulated in cancer tissues and participates in progression of cancer, including lung cancer, prostate cancer, breast cancer, and esophagogastric junction adenocarcinoma. STAT3-, β-catenin- and c-Myc- mediated signaling pathways work on downstream of HN1L (Jiao et al. 2021; Li et al. 2017; Wang et al. 2021). Our cell viability analysis supports the oncogenic role of HN1L in GC for knockdown HN1l suppressed GC cell growth. qRT-PCR and western blot results exhibited that HN1L was obviously influenced with ectopic expression or knockdown of METTL13. Besides, the increased growth rate induced by METTL13 overexpression was partially attenuated by HN1L silencing. These observations strongly suggest that HN1L is required for METTL13 induced cancer development. More interestingly, a recent report has shown that METTL13 also serves as a crucial mediator of HN1L in modulating hepatocellular carcinoma cell growth and metastasis via interacting with AP-2γ, raising a possibility that METTL13 and HN1L forms a positive feedback regulatory loop. Subsequent investigations via qRT-PCR in GC cells proved the possibility.

With regard to eEF1A, the methylation substrate of METTL13, studies have implicated its regulatory roles in the cellular biological process and pathological process (Abbas et al. 2015; Lamberti et al. 2004). Of notes, eEF1A1 and eEF1A2 were in connection with translation elongation and non-translational functions (Kahns et al. 1998; Soares and Abbott 2013). Distinct lysine residues, Lys36, Lys55, Lys79, Lys165 and Lys318 can be extensively methylated. METTL21B affected mRNA translation mainly due to methylation of eEF1A at Lys165. METTL13-mediated dimethylation of eEF1A at lysine55 contributes to protein synthesis in tumorigenesis (Cavallius et al. 1993; Hamey et al. 2016, 2017; Jakobsson et al. 2017; Malecki et al. 2017; Shimazu et al. 2014). From our experiments, no obvious expression alteration of HN1L was observed upon overexpressing mutant eEF1A from Lys 55 to Ala in GC cells. Therefore, further studies are needed to explore how METTL13 has an effect on eEF1A to promote HN1L expression.

In conclusion, we identified a new METTL13/eEF1A/HN1L signaling loop contributing to GC cell proliferation and metastasis. METTL13 may become a promising diagnostic and therapeutic biomarker for gastric cancer in the future.

## Supplementary Information

Below is the link to the electronic supplementary material.

Supplementary Fig. 1 **a** MRPS14 and SHQ1 mRNA expression was detected respectively upon silencing or overexpressing METTL13 in BGC823 and AGS cells. **b** Knockdown of HN1L decreased cell growth in AGS and MGC803 cells while overexpression of HN1L promoted cell growth in SGC7901 cells. **c** HN1L expression was examined in 48 pairs of GC samples via qRT-PCR. **d** Correlation between METTL13 and HN1L expression was statistically analyzed using *Chi*-square test based on qRT-PCR results of 48 pairs of GC samples. Data are given as mean ± SD. *P < 0.05; **P < 0.01.

Supplementary Fig. 2 **a** The relative intensity of protein (Flag-eEF1A/β-actin or HN1L/β-actin). **b–c** Cell growth was assessed by CCK-8 assay in GC cell lines transfected with siRNA or plasmids for eEF1A knockdown or overexpression. **d** METTL13 mRNA expression was measured upon upregulating or downregulating HN1L via qRT-PCR. Data are given as mean ± SD. *P < 0.05; **P < 0.01.

Supplementary Table 1 siRNAs and primers used in this study.

## Data Availability

All data analyzed and generated in this study are included in this published article and its supplementary information files.

## Abbreviations

GC: Gastric cancer
METTL13: Methyltransferase like 13
HCC: Hepatocellular carcinoma
TCF3: Transcription factor 3
ZEB1: Zinc finger E-box binding homeobox 1
HN1L: Hematological and neurological expressed 1-like
TMA: Tissue microarray
DMEM: Dulbecco’s modified Eagle’s
FBS: Fetal bovine serum
qRT-PCR: Quantitative real-time PCR
CCK-8 assay: Cell counting kit-8 assay
H&E: Hematoxylin and eosin
GEPIA: Gene expression profiling interactive analysis
TCGA: The cancer genome atlas
IHC: Immunohistochemical staining
eEF1A: Eukaryotic translation elongation factor-1A
MRPS14: Mitochondrial ribosomal protein S14
SHQ1: H/ACA ribonucleoprotein assembly factor
OD: Optical density
ANOVA: One-way analysis of variance
SD: Standard deviation
