# Supplementary figures and images for: METTL13 facilitates cell growth and metastasis in gastric cancer via an eEF1A/HN1L positive feedback circuit
Source: J Cell Commun Signal. 2022 Aug 4;17(1):121–35. doi: 10.1007/s12079-022-00687-x (PMC10030728; doi:10.1007/s12079-022-00687-x)

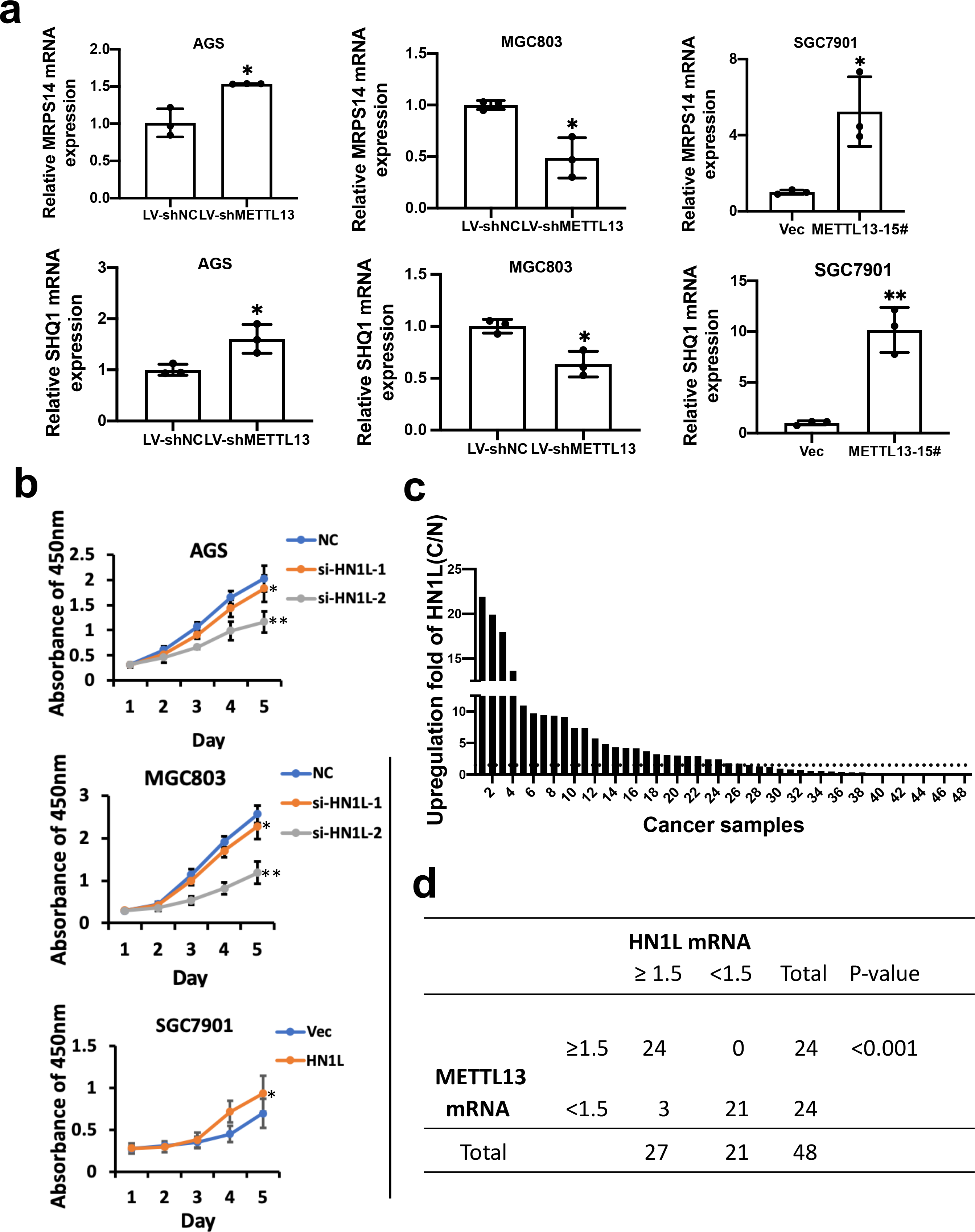

Supplement: Supplementary file 1 — Supplementary Fig. 1 a MRPS14 and SHQ1 mRNA expression was detected respectively upon silencing or overexpressing METTL13 in BGC823 and AGS cells. b Knockdown of HN1L decreased cell growth in AGS and MGC803 cells while overexpression of HN1L promoted cell growth in SGC7901 cells. c HN1L expression was examined in 48 pairs of GC samples via qRT-PCR. d Correlation between METTL13 and HN1L expression was statistically analyzed using Chi-square test based on qRT-PCR results of 48 pairs of GC samples. Data are given as mean ± SD. *P < 0.05; **P < 0.01. [file 12079_2022_687_MOESM1_ESM.tif]

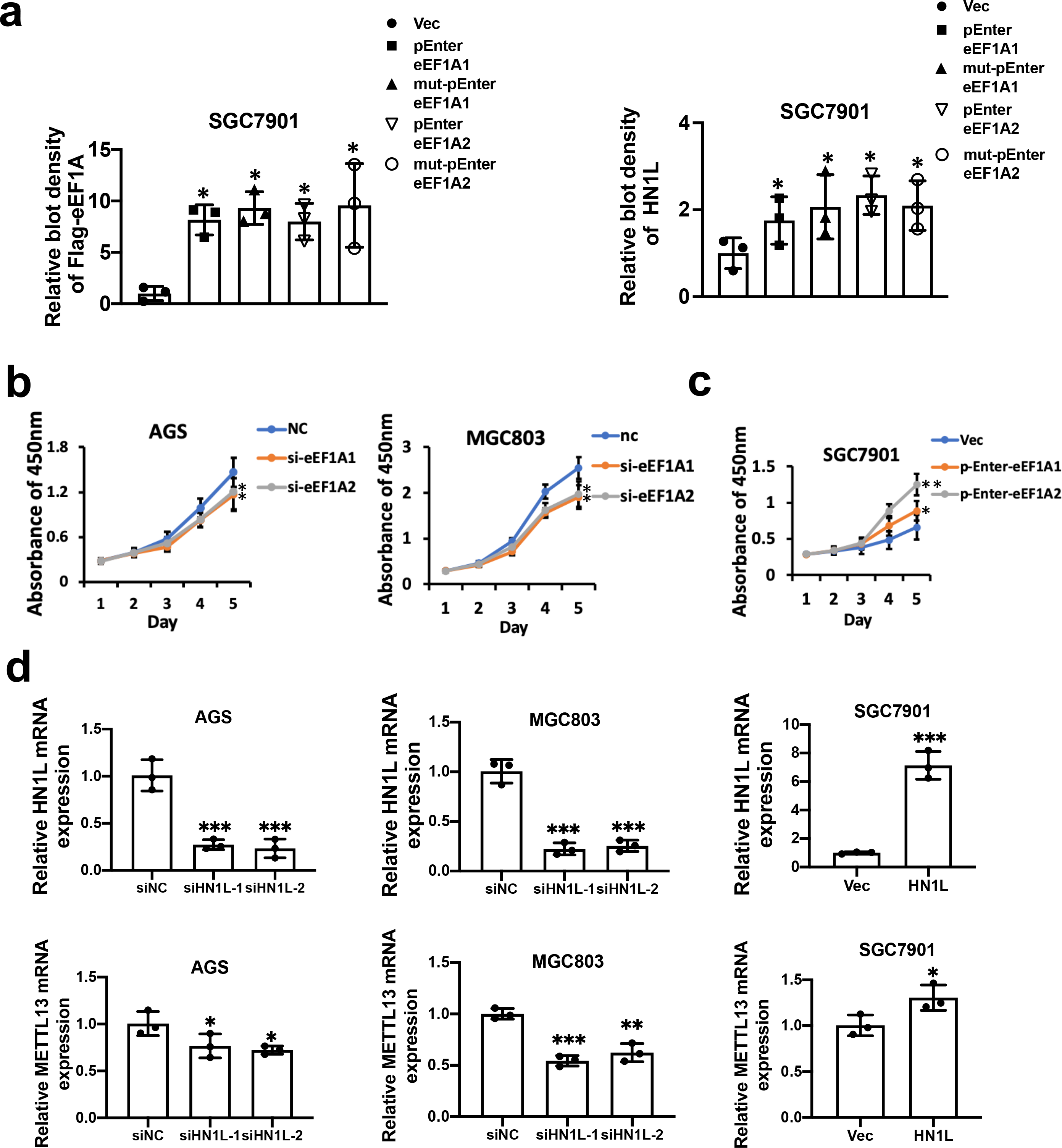

Supplement: Supplementary file 2 — Supplementary Fig. 2 a The relative intensity of protein (Flag-eEF1A/β-actin or HN1L/β-actin). b–c Cell growth was assessed by CCK-8 assay in GC cell lines transfected with siRNA or plasmids for eEF1A knockdown or overexpression. d METTL13 mRNA expression was measured upon upregulating or downregulating HN1L via qRT-PCR. Data are given as mean ± SD. *P < 0.05; **P < 0.01. [file 12079_2022_687_MOESM2_ESM.tif]
