# Supplementary material for: METTL13 facilitates cell growth and metastasis in gastric cancer via an eEF1A/HN1L positive feedback circuit
Source: J Cell Commun Signal. 2022 Aug 4;17(1):121–35. doi: 10.1007/s12079-022-00687-x (PMC10030728; doi:10.1007/s12079-022-00687-x)
Supplement: Supplementary file 3 — Supplementary Table 1 siRNAs and primers used in this study. [file 12079_2022_687_MOESM3_ESM.docx]

**Supplementary table 1: siRNAs and primers used in this study**

| **siRNA sequences (sense):** |
| --- |
| siNC: 5’-UUCUCCGAACGUGUCACGUdTdT-3’ |
| siMETTL13-1: 5’-GCGGGGUGCUACAUAAAUAdTdT-3’ |
| siMETTL13-2: 5’-GGGCAGUCCAUUGAUAAGAdTdT-3’ |
| siHN1L-1: 5’-CCAAGGAUCAUGUUUUCUUdTdT-3’ |
| siHN1L-2: 5’-CCUCAGAACAUACCCAAGAdTdT-3’ |
| sieEF1A1: 5’-AUGCGGUGGCAUCGACAAAdTdT-3’ |
| sieEF1A2: 5’-UGGUCCUUUUGUCAAUACCUCCGCA-3’ |
| **Primer sequences for qRT-PCR:** |
| β-actin-qF: 5’-CCTGGCACCCAGCACAATG-3’  β-actin-qR: 5’-GGGCCGGACTCGTCATACT-3’ |
| METTL13-qF: 5’-GCAGGAGGAGGAGAAGCACG-3’  METTL13-qR: 5’-GGTTGAGAATAAAAACACCTTCAGG-3’ |
| HN1L-qF: 5’-CCCAGGAGGAGAATCGAGCA-3’  HN1L-qR: 5’-CGGGGGTTGATTCGTCAAAG-3’ |
| eEF1A1-qF: 5’-AACATTGTCGTCATTGGACA-3’  eEF1A1-qR: 5’-ACTTGCTGGTCTCAAATTTC-3’ |
| eEF1A2-qF: 5’-GAAGGTGGAGCGTAAGGAGG-3’  eEF1A2-qR: 5’-AAAGGTCACCACCATGCCC-3’ |
| SHQ1-qF: 5’-GTACTTCGAGGGGGTGGACT-3’  SHQ1-qR: 5’-CATAGGTCCCCTGCTCAGAT-3’ |
| MRPS14-qF: 5’-GGACGTTCAAGCAGATGGT-3’  MRPS14-qR: 5’-CTTCATCAGCCACATCCTGA-3’ |
